# Clinical Presentations of Chagas Cardiomyopathy

**DOI:** 10.1155/2020/8884910

**Published:** 2020-11-03

**Authors:** Malik Shehadeh, Ana Barac, Rachel Marcus

**Affiliations:** ^1^Medstar Washington Hospital Center, Washington, D. C, USA; ^2^Medstar Heart and Vascular Institute, Washington, D. C, USA; ^3^Medstar Union Memorial Hospital and Latin American Society of Chagas (LASOCHA), USA

## Abstract

Chronic Chagas cardiomyopathy (CCC) is the most common cause of nonischemic cardiomyopathy in endemic Latin American countries. Immigrants to the United States suffer from this disease, but it is underrecognized. We describe the three hallmark clinical presentations: stroke, ventricular arrhythmias, and heart failure, which should prompt suspicion for CCC.

## 1. Introduction

Chagas disease is a chronic systemic parasitic infection caused by the protozoan Trypanosoma cruzi that is transmitted to humans by triatomine insects in endemic areas of Latin America [1].

CCC typically appears 15-30 years after the initial infection, and 20-30% of infected individuals present with manifestations such as heart failure, stroke, and brady- and tachyarrhythmias [1–3]. Because of lack of awareness of this disease on the part of US-based health care professionals, the diagnosis is underrecognized.

In this case series, we describe the experience of a high-volume referral center with different presentations of Chagas cardiomyopathy, highlighting presentations that should raise suspicion for Trypanosoma cruzi infection.

## 2. First Presentation: Stroke

A 28-year-old El Salvadorian male was referred to cardiology for palpitations after an electrocardiogram (ECG) showed bifascicular block. A transthoracic echocardiogram (TTE) showed normal biventricular size and function, and a cardiac magnetic resonance imaging (MRI) was suggested because of the ECG abnormalities. This showed a small area of transmural myocardial fibrosis with focal dyskinesis of the true apex, muscular diverticula, and midapical lateral hypokinesis. Because of the myocardial scar, a coronary computed tomography angiography was suggested to evaluate for coronary atherosclerosis, but the patient was lost to follow-up.

**Figure 1:**
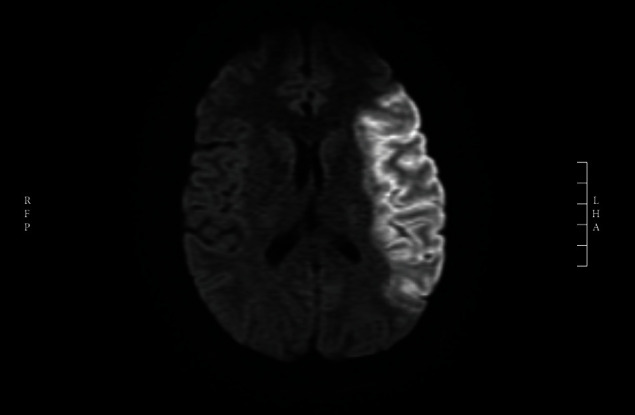
Diffusion-weighted brain magnetic resonance imaging (MRI) showing acute stroke in the left middle cerebral artery territory.

2 years later, he presented with new right hemiparesis of indeterminate duration. Brain MRI revealed acute ischemic infarct in the left middle cerebral artery territory (Figure 1). Thrombolytic therapy was not considered given the unknown onset of stroke, and he was admitted to the hospital for further ischemic workup.

**Figure 2:**
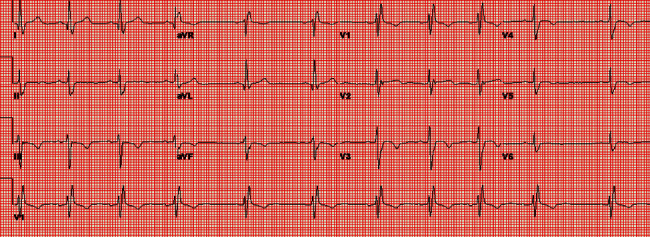
Electrocardiogram showing normal sinus rhythm with right bundle branch block, left anterior fascicular block, and secondary T wave changes.

Electrocardiogram (ECG) showed normal sinus rhythm with right bundle branch block (RBBB), left anterior fascicular block (LAFB), and secondary T wave changes (Figure 2). TTE with contrast showed normal left ventricular ejection fraction (LVEF), and with echo contrast injection, an apical aneurysm was noted.

Given the patient's country of origin, ECG, and TTE findings, CCC was considered. Commercial Hemagen followed by CDC confirmatory assays were positive for Trypanosoma cruzi infection, which confirmed the diagnosis.

Patient was discharged to a rehabilitation facility and continued to follow up with our clinic. Family screening was discussed as an outpatient.

## 3. Second Presentation: Ventricular Tachycardia

A 46-year-old El Salvadorian female with a history of alcohol abuse presented to our hospital with hemodynamically unstable ventricular tachycardia requiring cardioversion.

**Figure 3:**
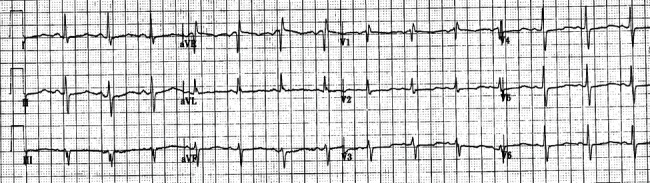
Electrocardiogram showing normal sinus rhythm with bifascicular block (right bundle branch block and left anterior fascicular block).

She presented with chest tightness and dizziness of 2-hour duration and was found to be in monomorphic ventricular tachycardia requiring cardioversion. Laboratory investigations showed low potassium and magnesium levels which were repleted. Thyroid function was normal. Troponin I was 0.581 ng/ml (reference range < 0.045 ng/ml). ECG after cardioversion showed normal sinus rhythm with RBBB and LAFB (Figure 3).

**Figure 4:**
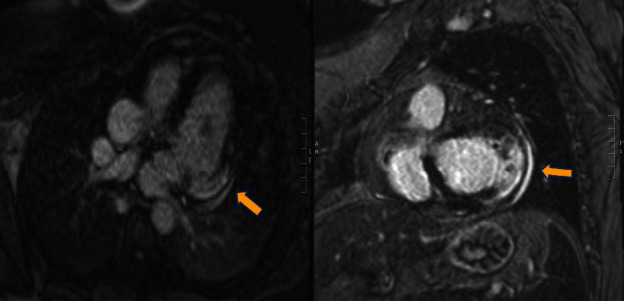
Cardiac magnetic resonance imaging (MRI) showing transmural late gadolinium enhancement in the basal lateral wall (yellow arrows) which is highly concerning of Chagas cardiomyopathy.

Coronary angiography showed no coronary artery disease. TTE showed normal biventricular function without regional wall motion abnormalities. Cardiac MRI showed extensive transmural basal lateral wall gadolinium enhancement suggestive of fibrosis (Figure 4). Commercial Trypanosoma cruzi enzyme-linked immunosorbent assay (ELISA) followed by CDC serologic testing confirmed the diagnosis of CCC.

The patient received a single-chamber implantable cardioverter defibrillator for secondary prevention. Family screening was discussed as an outpatient; the patient was made aware that all children of an infected mother must receive testing to evaluate for congenital transmission.

## 4. Third Presentation: Heart Failure

A 43-year-old El Salvadorian male with heart failure from CCC required left ventricular assist device (LVAD) implantation and subsequently orthotopic heart transplantation.

The patient presented at the age of 22 with a syncopal event for which a dual-chamber pacemaker was placed. After developing symptoms of heart failure in his early 30's, he was found by TTE to have severe systolic dysfunction with an ejection fraction of 10%. Coronary angiography showed no obstructive coronary artery disease. Nonischemic lab analysis was unrevealing.

He underwent right heart catheterization with biopsy which showed multifocal chronic inflammation with fibrosis and myocardial necrosis. Given his country of origin, there was high suspicion of Chagas disease as the precipitating cause of his nonischemic cardiomyopathy. Trypanosoma cruzi serologic assays were obtained and were positive.

Despite guideline-directed medical therapy, he required inotropic support and subsequently a HeartWare LVAD was placed. Two years later, the patient received a heart transplant and within 2 weeks of transplant was diagnosed with acute reactivation of Chagas by polymerase chain reaction assay. He responded well to benznidazole therapy.

## 5. Discussion

Chagas Cardiomyopathy is the most important clinical presentation of Chagas disease. While only 20-30% of infected patients will develop cardiomyopathy [3], those who do will have a spectrum of findings ranging from abnormal ECG alone to severe biventricular dysfunction and even sudden cardiac death. The three major clinical presentations of Chagas cardiomyopathy include arrhythmias, heart failure, and thromboembolism [1, 3].

ECG findings that are highly suggestive of Chagas cardiomyopathy include first-degree atrioventricular block, RBBB, LAFB, bifascicular block, or premature ventricular contractions [3]. Nevertheless, the absence of these findings does not mean the diagnosis is not present. Hallmark echocardiographic features include regional wall motion abnormalities, particularly of the basal inferolateral wall, and an apical aneurysm/thrombus that can be difficult to image without contrast or cardiac MRI, especially when the remainder of the left ventricle is normal [4]. Gadolinium uptake on cardiac MRI is frequent and more pronounced as LVEF decreases. Studies demonstrate that gadolinium uptake correlates well with the risk of arrhythmias [4].

Patients with Chagas cardiomyopathy with preserved or mildly depressed ejection fraction are still at risk for lethal ventricular arrhythmias as well as strokes, and strong consideration should be given to risk to stratify those patients with cardiac imaging [3, 4].

All children of seropositive women and any additional family members of an infected individual should be screened for the disease because of the risk of maternal-fetal transmission and the higher rates of Chagas disease in immediate family members [2, 6].

Most importantly, physicians in the United States who work with Latin American immigrants should be aware that this disease in not uncommon in patients presenting with unexplained cardiac symptoms and/or suspicious electrocardiographic features. Seroprevalence studies have shown high rates of Chagas disease in Latin American immigrants from Chagas-endemic countries to the U.S.: at the Olive View Medical Center in Los Angeles County, 5% of patients with conduction abnormalities [7], 7.5% of patients with pacemakers [9], 18% of patients with bifascicular block [7], and 19% of patients with nonischemic cardiomyopathy were found to have Chagas disease [8], and at Elmhurst hospital in the Bronx, 13% of nonischemic cardiomyopathy patients were found to have Chagas disease [5].

## 6. Conclusion

Chagas disease should be strongly suspected in Latin American immigrant patients from endemic countries who present with stroke, ventricular arrhythmias, or heart failure.

## Abbreviations

CCC: Chronic Chagas cardiomyopathy
ECG: Electrocardiogram
TTE: Transthoracic echocardiogram
MRI: Magnetic resonance imaging
RBBB: Right bundle branch block
LAFB: Left anterior fascicular block
LVEF: Left ventricular ejection fraction
CDC: Centers for Disease Control and Prevention
ELISA: Enzyme-linked immunosorbent assay
LVAD: Left ventricular assist device.

## Additional Points

*Learning Objectives*. (1) Chagas cardiomyopathy can present with different clinical presentations. The three hallmark clinical presentations are stroke, ventricular arrhythmias, and heart failure. (2) Chagas cardiomyopathy should be suspected in Latin American immigrant patients from endemic countries who present with unexplained cardiac symptoms and/or suspicious ECG features. (3) Chagas cardiomyopathy can have serious manifestations even in the presence of normal LVEF.
